# Circulating long chain acylcarnitines and outcomes in diabetic heart failure: an HF-ACTION clinical trial substudy

**DOI:** 10.1186/s12933-021-01353-z

**Published:** 2021-08-03

**Authors:** Lauren K. Truby, Jessica A. Regan, Stephanie N. Giamberardino, Olga Ilkayeva, James Bain, Christopher B. Newgard, Christopher M. O’Connor, G. Michael Felker, William E. Kraus, Robert W. McGarrah, Svati H. Shah

**Affiliations:** 1 Duke Molecular Physiology Institute, Duke University Medical Center, Durham, NC 27710 USA; 2 Department of Medicine, Division of Cardiology, Duke University School of Medicine, Durham, NC USA; 3 Inova Heart and Vascular Institute, Falls Church, NC USA; 4 Precision Genomics Collaboratory, Duke University School of Medicine, Durham, USA

**Keywords:** Heart failure, Diabetes, Exercise, Long chain acylcarnitines

## Abstract

**Background:**

Whether differences in circulating long chain acylcarnitines (LCAC) are seen in heart failure (HF) patients with and without diabetes mellitus (DM), and whether these biomarkers report on exercise capacity and clinical outcomes, remains unknown. The objective of the current study was to use metabolomic profiling to identify biomarkers that report on exercise capacity, clinical outcomes, and differential response to exercise in HF patients with and without DM.

**Methods:**

Targeted mass spectrometry was used to quantify metabolites in plasma from participants in the heart failure: a controlled trial investigating outcomes of exercise training (HF-ACTION) trial. Principal components analysis was used to identify 12 uncorrelated factors. The association between metabolite factors, diabetes status, exercise capacity, and time to the primary clinical outcome of all-cause mortality or all-cause hospitalization was assessed.

**Results:**

A total of 664 participants were included: 359 (54%) with DM. LCAC factor levels were associated with baseline exercise capacity as measured by peak oxygen consumption (beta 0.86, p  =  2 × 10^−7^, and were differentially associated in participants with and without DM (beta 1.58, p  =  8  ×  10^−8^ vs. 0.67, p  =  9  ×  10^−4^, respectively; p value for interaction  =  0.012). LCAC levels changed to a lesser extent in participants with DM after exercise (mean ∆ 0.09, p  =  0.24) than in those without DM (mean ∆ 0.16, p  =  0.08). In univariate and multivariate modeling, LCAC factor levels were associated with time to the primary outcome (multivariate HR 0.80, p  =  2.74  ×  10^−8^), and were more strongly linked to outcomes in diabetic participants (HR 0.64, p  =  3.21  ×  10^−9^ v. HR 0.90, p  =  0.104, p value for interaction  =  0.001). When analysis was performed at the level of individual metabolites, C16, C16:1, C18, and C18:1 had the greatest associations with both exercise capacity and outcomes, with higher levels associated with worse outcomes. Similar associations with time to the primary clinical outcome were not found in a control group of patients without HF from the CATHeterization GENetics (CATHGEN) study.

**Conclusions:**

LCAC biomarkers are associated with exercise status and clinical outcomes differentially in HF patients with and without DM. Impaired fatty acid substrate utilization and mitochondrial dysfunction both at the level of the skeletal muscle and the myocardium may explain the decreased exercise capacity, attenuated response to exercise training, and poor clinical outcomes seen in patients with HF and DM.

*Trial Registration* clinicaltrials.gov Identifier: NCT00047437.

**Supplementary Information:**

The online version contains supplementary material available at 10.1186/s12933-021-01353-z.

## Background

Heart failure (HF) is becoming an increasingly common problem in the aging population: 6.2 million Americans are currently living with HF and it is estimated that by 2030 HF will affect almost 3% of the population [1]. The heart failure: a controlled trial investigating outcomes of exercise training (HF-ACTION) study was a randomized controlled trial of 2331 ambulatory patients with HF that assessed functional capacity and the response to exercise training, demonstrating the benefit of exercise in patients with HF with reduced ejection fraction [2]. However, there was heterogeneity in the response to exercise training, particularly in patients with diabetes mellitus (DM) that was not understood at a molecular level.

Participants in the HF-ACTION trial with comorbid diabetes (32%) demonstrated significantly lower baseline peak oxygen consumption (pVO_2_) and 6-min walk test distance as compared to patients without comorbid diabetes. Additionally, DM appeared to attenuate the benefit of exercise training as measured by pVO_2_ even after adjustment for exercise volume [3]. These data add to the known, complex relationship between DM and HF, where the incidence of HF is greater than two-fold higher in patients with DM than in sex-matched controls, and the prevalence of cardiomyopathy in patients with DM has been reported to be as high as 35% [4, 5]. Although the clinical intersection of these cardiometabolic conditions has been well-characterized, the biochemical basis for this association remains poorly understood.

The heart is a metabolic machine with the greatest energy requirements of any organ, relying on adequate energy substrate delivery from peripheral tissues and efficient intrinsic metabolism to ensure optimal cardiac health [6]. As such, disruption of metabolic processes in peripheral tissues such as skeletal muscle and liver and in the heart itself are key early events underlying the pathophysiology of most cardiac diseases [7]. Both HF and DM independently lead to alterations in energy homeostasis and impaired cardiac and systemic metabolism; however, whether a unique metabolic signature exists in the presence of comorbid HF and DM has not been determined.

Metabolomic profiling is an effective scientific tool that can be used to simultaneously identify metabolic molecular mechanisms and biomarkers of HF [8–10]. In particular, it has previously been shown that accumulation of circulating long-chain acylcarnitines (LCAC), byproducts of oxidation of fatty acids and the primary fuel source in the normal heart, are associated with outcomes in HF and are reversible with introduction of mechanical support [11]. Thus, the primary objectives of the current study were to (1) assess the relationship between LCAC levels and DM status in patients with HF; (2) to determine whether LCAC levels were associated with baseline exercise capacity as measured by pVO_2_ in those with and without DM; and (3) to quantify the relationship between baseline LCAC levels and time to the primary outcome of all-cause mortality or hospitalization.. We leveraged the HF-ACTION biospecimen repository with carefully adjudicated intermediate and hard outcomes to assess these pathways and biomarkers.

## Methods

### Study population

The population for this study consisted of 666 participants from the HF-ACTION clinical trial who had previously consented to have samples of peripheral blood collected for future studies. Two patients with missing clinical covariates were excluded from association testing. Overall details of participants in HF-ACTION have been described previously [2]. Briefly, HF-ACTION enrolled participants who were at least 18 years of age and were known to have ambulatory HF with a left ventricular ejection fraction (LVEF) of  <  35%. Participants were randomized to usual HF care versus usual care plus a supervised exercise training program with 3 group-based, sessions each week for 12 weeks followed by a home exercise program. Measures of exercise capacity including 6-min walk test distance and pVO_2_ from a cardiopulmonary exercise test were assessed at baseline and at three months after study enrollment. The primary clinical outcome of interest of the HF-ACTION clinical trial was the composite endpoint of all-cause mortality or all-cause hospitalization. To create a control group, we also leveraged the available metabolomic data in the CATHeterization GENetics (CATHGEN) to assess the association between individual LCAC levels and the outcome of all-cause mortality in this control group without prevalent HF.

### Laboratory methods

As has been previously described, targeted metabolomics were performed in two batches using a quantitative, tandem-flow injection mass spectrometry-based approach to assess levels of 45 acylcarnitines and 15 amino acids in stored, frozen, previously unthawed plasma samples from the HF-ACTION biorepository. Samples were collected under the standardized study protocol in a fasting state. Baseline metabolites were measured in 666 participants and metabolites measured 3 months after enrollment in 537 participants. Proteins were removed by precipitation and aliquoted supernatants were dried. Acylcarnitines were esterified with hot acidic methanol and amino acids esterified with n-butanol [12]. Tandem mass spectrometry was used for the analysis with a Quattro Micro instrument (Waters Corp., Milford, Massachusetts); addition of internal standards enabled quantitative assessment of metabolites. All assays were done by the Metabolomics Core Laboratory of the Duke Molecular Physiology Institute at Duke University. Personnel were blinded to clinical characteristics of the patients as well as treatment group.

### Statistical analysis

Analyses were performed to assess (1) comparison of baseline characteristics between patients with and without diabetes, (2) association of baseline metabolomic factors with diabetes status, (3) baseline metabolites and exercise capacity, and (4) the relationship between baseline factors and clinical outcomes. All outcomes were stratified by diabetes status, which was assessed at the time of study enrollment based upon self-report and was later confirmed by a clinician based upon available medical history and laboratory data. First, for analysis 1, baseline clinical characteristics were compared between diabetic and non-diabetic patient groups using chi-square tests for categorical variables and Kruskal–Wallis rank sum tests for continuous variables. All clinical covariates’ distributions and missingness were assessed, and 2 patients with significant outlier values for their baseline creatinine were excluded from the analysis. For analysis 2–4, principal components analysis (PCA) with varimax rotation was performed using baseline metabolites to reduce the large number of correlations between metabolites into 12 uncorrelated factors meeting the Kaiser criterion of having an eigenvalue  >  1. This approach has been used previously [12]. Three-month metabolite factor scores were calculated for individuals by projecting baseline factor weights onto 3-month metabolite data that had been scaled to baseline data. For analysis 2 and 3, generalized linear models were used to evaluate the association between PCA-derived factors, diabetes status and baseline pVO_2_, baseline duration of exercise during cardiopulmonary exercise (CPX) testing, and baseline six-minute walk distance (6MWD). For analysis 4, Cox proportional hazards models were generated for the primary outcome of all-cause mortality or hospitalization. Proportional hazards assumptions were tested and verified at a p value of 0.05. Multivariate models were adjusted for age, baseline LVEF, sex, race, baseline creatinine, body mass index (BMI), peak VO2, BUN, CPX duration, and treatment group. After univariate analyses were performed, all subsequent multivariate, DM-stratified and interaction analyses were performed for the subset of factors that exhibited a Benjamini & Hochberg (BH) p value  <  0.05 in univariate analyses, in order to control for multiple comparisons during the initial univariate analyses. Although BH p values  <  0.05 were considered statistically significant and used for performing downstream analyses after initial testing, nominal p values are presented for subsequent analyses. For those factors that were significantly associated with exercise capacity and/or time to primary events, individual metabolites with an absolute factor loading of  >  0.4 were analyzed to identify those with the greatest effect. This time to event analysis was replicated in participants without HF that were enrolled in the CATHGEN study and had available metabolomics data to create a control group for the primary outcome of all-cause mortality. Kaplan Meier survival univariate estimates were generated for time to event analyses, stratified by quartiles of factor level for relevant factors. Natural log transformed metabolite levels were utilized in univariate and multivariate analyses. All reported beta coefficients are unstandardized.

## Results

### Baseline participant characteristics

Baseline study participant characteristics are presented in Table 1. Among 664 analyzed participants, 359 (54.1%) had previously been given a clinical diagnosis of Type 2 DM and 183 (27.6%) were female. The median age of the population was 60 years [interquartile range (IQR) 52.6–68.1] with participants with DM being older [median age 61.1 years (IQR 54.6–68.1) v. 58.5 (IQR 50.1–68.2), p  =  0.01] and having a higher median BMI [32.0 (IQR 27.8–37.3) v. 29.8 (IQR 25.6–34.4), p  <  0.001]. Thirty-six (10.1%) of participants with DM were current smoker as compared to 59 (19.5%) without DM (p  =  0.001). The median baseline pVO_2_ was 14.0 (IQR 11.2–16.9) in the overall cohort and lower in participants with DM as compared with those without DM [13.4 (IQR 10.4–16.6) v. 14.8 (IQR 11.9–17.7), p  <  0.001]. There was no significant difference between baseline NT-proBNP or LVEF between groups.

**Table 1:** Baseline patient characteristics

Variable	Overall (n = 664)	Non-diabetic (n = 305)	Diabetic (n = 359)	p-value
Clinical variables				
Age (years), [median(IQR)]	60.0 [52.6, 68.1]	58.5 [50.1, 68.2]	61.1 [54.6, 68.1]	**0.010**
Female sex (%)	183 (27.6)	93 (30.5)	90 (25.1)	0.141
Race (%)				
American Indian	5 (0.8)	0 (0.0)	5 (1.4)	**0.030**
Asian	9 (1.4)	1 (0.3)	8 (2.2)	
African American	213 (32.1)	97 (31.8)	116 (32.3)	
White	410 (61.7)	195 (63.9)	215 (59.9)	
Multiracial	15 (2.3)	9 (3.0)	6 (1.7)	
Unknown	12 (1.8)	3 (1.0)	9 (2.5)	
BMI [median (IQR)]	30.7 [26.8, 36.1]	29.8 [25.6, 34.4]	32.0 [27.8, 37.3]	**< 0.001**
Pulse pressure (mmHg), [median (IQR)]	42.0 [36.0, 54.0]	40.0 [34.0, 50.0]	46.0 [37.2, 56.0]	**< 0.001**
History of atrial arrhythmia (%)	142 (21.4)	62 (20.3)	80 (22.3)	0.605
Currently smoking (%)	95 (14.4)	59 (19.5)	36 (10.1)	**0.001**
LVEF (%) [median (IQR)]	25.0 [20.0, 30.0]	25.0 [19.6, 30.1]	25.0 [20.0, 29.9]	0.510
NT-Pro BNP [median (IQR)]	813.0 [342.5, 1859.0]	751.9 [320.8, 1854.0]	899.1 [377.5, 1855.2]	0.252
Hemoglobin (g/dL) [median (IQR)]	13.3 [12.1, 14.4]	13.3 [12.3, 14.5]	13.1 [12.0, 14.3]	0.076
Creatinine (mg/dL) (median [IQR])	1.2 [1.0, 1.5]	1.1 [1.0, 1.4]	1.3 [1.0, 1.6]	**< 0.001**
Hemoglobin A1C [median (IQR)]	6.9 [6.2, 8.0]	5.9 [5.5, 6.3]	7.2 [6.4, 8.4]	**< 0.001**
BUN (mg/dL) [median (IQR)]	21.0 [15.0, 29.0]	18.0 [14.0, 24.0]	24.0 [17.0, 33.0]	**< 0.001**
ACEi dose (mg) [median (IQR)]	20.0 [0.0, 40.0]	20.0 [0.0, 40.0]	20.0 [0.0, 40.0]	0.610
Beta blocker dose (mg) [median (IQR)]	25.0 [13.0, 50.0]	25.0 [13.0, 50.0]	50.0 [16.0, 50.0]	**0.008**
Exercise variables				
Baseline peak VO_2_ (mg/kg/min) [median (IQR)]	14.0 [11.2, 16.9]	14.8 [11.9, 17.7]	13.4 [10.4, 16.6]	**< 0.001**
6-minute walk distance (m) [median (IQR]]	361.0 [289.7, 426.3]	365.8 [305.4, 435.1]	353.0 [274.7, 415.6]	**0.012**

*BMI* body mass index; *LVEF* left ventricular ejection fraction; *BNP* brain natriuretic peptide; *BUN* blood urea nitrogen; *ACEi* angiotensin converting enzyme inhibitor; *VO*_*2*_, oxygen consumption

Bold values indicate a statistically significance difference in P-values

### Metabolomic factors are associated with baseline DM status

PCA identified 12 metabolite factors, each consisting of metabolites that clustered in shared biological pathways (Table 2), similar to our prior studies [12]. Factor 2 (long-chain dicarboxylacylcarnitines), factor 3 (short chain acylcarnitines), factor 6 (amino acids dominated by glycine), and factor 7 (Met, C5:1, C22, C16:1-OH/C14:1-DC) were all significantly associated with baseline diabetic status after FDR adjustment for multiple comparisons. Interestingly, factor 12, composed of branched chain amino acids (BCAA) and aromatic amino acids, which have previously been associated with insulin resistance and DM, was not associated with baseline DM status (nominal p  =  0.06, FDR p  =  0.1).

**Table 2:** Principal components factors and their association with diabetic status

Factor	Loading	Description	Metabolites with absolute loading > 0.4	Diabetes beta	p value	BH p value
1	(−)	Medium and long chain acylcarnitines	C8, C10:1, C10, C12:1, C12, C14:2, C14:1, C14, C16, C18:2, C18:1, C18, C16:2, C16:1	0.138	0.076	0.102
2	(+)	Long chain dicarboxylacylcarnitines	C10-OH/C8-DC, C12-OH/C10-DC, C14:1-OH/C12:1-DC, C14-OH/C12-DC, C16-OH/C14-DC, C18:1-OH/C16:1-DC, C18-OH/C16-DC, C20, C18:1-DC, C20-OH/C18-DC, C22	**0.454**	**4E-9**	**2E-8**
3	(+)	Short chain acylcarnitines	C2, C3, C4/Ci4, C5's, C5-OH/C3-DC	**0.214**	**0.006**	**0.017**
4	(+)	Medium chain dicarboxylacylcarnitines	Cit, C5-OH/C3-DC, Ci4-DC/C4-DC, C5-DC, C6-DC, C10-OH/C8-DC, C6:1-DC/C8:1-OH, C8:1-DC	0.138	0.022	0.053
5	Mixed	Long chain acylcarnitines	Arg (+), C16(−), C18:2(−), C18:1(−), C18(−), C20:4(−)	− 0.012	0.875	0.875
6	(−)	Amino acids	Gly, Ser, Arg	**0.429**	**3E-8**	**1E-7**
7	(+)	Miscellaneous	Met, C5:1, C22, C16:1-OH/C14:1-DC	**1.067**	**1E-49**	**1E-48**
8	(−)	Medium chain acylcarnitines	C8:1, C10:3, C10:2, C10:1, C6:1-DC/C8:1-OH, C8:1-DC	− 0.128	0.097	0.116
9	(−)	Amino acids	Ala, Pro, His	− 0.141	0.069	0.102
10	Mixed	Amino acids	His(+), Asx(−), Arg(+)	− 0.092	0.234	0.255
11	(+)	Long chain dicarboxylacylcarnitines	C2, C4-OH, C14:1-OH/C12:1-DC	0.167	0.031	0.062
12	(+)	Branched chain amino acids|amino acids	Val, Leu/Ile, Phe, Tyr, Orn	− 0.147	0.057	0.098

P-values in bold indicate a statistically significant association

### Long-chain acylcarnitine (LCAC) metabolites are associated with exercise capacity and show an interaction with DM status

Several metabolite factors were associated with baseline pVO_2_ after FDR adjustment for multiple comparisons (Table 3) including factor 1 [medium chain acylcarnitines (MCAC) and LCAC], factor 4 [medium chain dicarboxylacylcarnitines (MCDA)] and factor 8 [MCAC and short dicarboxylacylcarnitines (SCDA)] (FDR p  =   <  0.001–0.02). Taking into account directionality of the beta and of factor loadings (Table 2), this shows that MCAC and LCAC (factor 1), MCAC and SCDA (factor 8), and individual SCDA (factor 4) are all negatively associated with pVO_2._ All factors meeting significance after FDR adjustment were then tested in stratified analysis by DM (Table 3) which showed that factors 1, 4, 5 and 8 were different between participants with and without DM. In formal analyses of a diabetes-factor interaction term, only factor 1 showed significant interaction effects with DM (p  =  0.012), with factor 1 levels more strongly associated with pVO_2_ in participants with DM as compared to those without (beta 1.58, p  =  8  ×  10^−8^ vs. 0.67, p  =  9  ×  10^−4^). Given this finding, we further explored the association between factor 1 and exercise capacity by examining CPX duration and 6MWD. Factor 1 was also associated with CPX duration (beta 0.744, p  =  2  ×  10^−7^) in the overall population, and appeared to have differential effects in participants with and without diabetes (beta 1.33, p  =  6  ×  10^−7^ v. beta 0.585, p  =  5  ×  10^−4^), suggesting that diabetic patients with higher MCAC/LCAC levels have shorter CPX duration given negative factor loading. Additionally, factor 1 was very strongly associated with 6MWD, with diabetic participants demonstrating a stronger association than those without diabetes (beta 45.1, p  =  3  ×  10^−9^). Because metabolites are negatively loaded in Factor 1, this suggests that lower levels of individual metabolites are associated with short 6MWD.

**Table 3:** Association of principal components factors with exercise capacity (pVO_2_)

Factor	Overall			Non-diabetic		Diabetic		Interaction p value
	Factor beta	p value	BH p value	Factor beta	p value	Factor beta	p value	
Baseline								
1	0.857	**2E-7**	**1E-6**	0.669	**9E-4**	1.58	**8E-8**	**0.012**
2	− 0.572	**− 0.001**	**− 0.001**	− 1.12	**− 0.013**	− 0.296	0.088	0.077
3	− 0.596	**4E-4**	**0.001**	− 0.351	0.233	− 0.615	**0.002**	0.443
4	− 1.194	**2E-8**	**3E-7**	1.43	**5E-5**	− 0.906	**5E-4**	0.222
5	0.623	**2E-4**	**0.001**	0.432	0.076	0.822	**2E-4**	0.234
6	− 0.215	0.198	0.238					
7	0.088	0.598	0.653					
8	0.773	**4E-6**	**1E-5**	0.562	0.065	0.811	**2E-5**	0.473
9	− 0.314	0.061	0.081					
10	0.035	0.837	0.837					
11	− 0.457	**0.006**	**0.011**	− 0.443	0.099	− 0.359	0.083	0.803
12	0.430	**0.011**	**0.017**	0.603	**0.034**	0.238	0.245	0.288

P-values in bold indicate a statistically significant association

In analyses of baseline metabolite factors with change in pVO_2_ between baseline and three months_,_ no factor was significant nominally nor after FDR adjustment for multiple comparisons (Additional file 2: Table S1), although factor 8 showed a trend for significance (nominal p  =  0.07) for baseline levels associated with change in pVO_2_. In addition, when linear mixed modeling including treatment group, timepoint (baseline v. 3 months), and an interaction term was performed with individual metabolite levels from Factor 4, 5, and 8 being the dependent variable, only C18 had a significant interaction term (p  =  0.02) suggesting that its levels decrease with exercise over time (Additional file 1: Figure S1).

### LCAC metabolites predict composite clinical outcome and show differential association by DM status

**Fig. 1:**
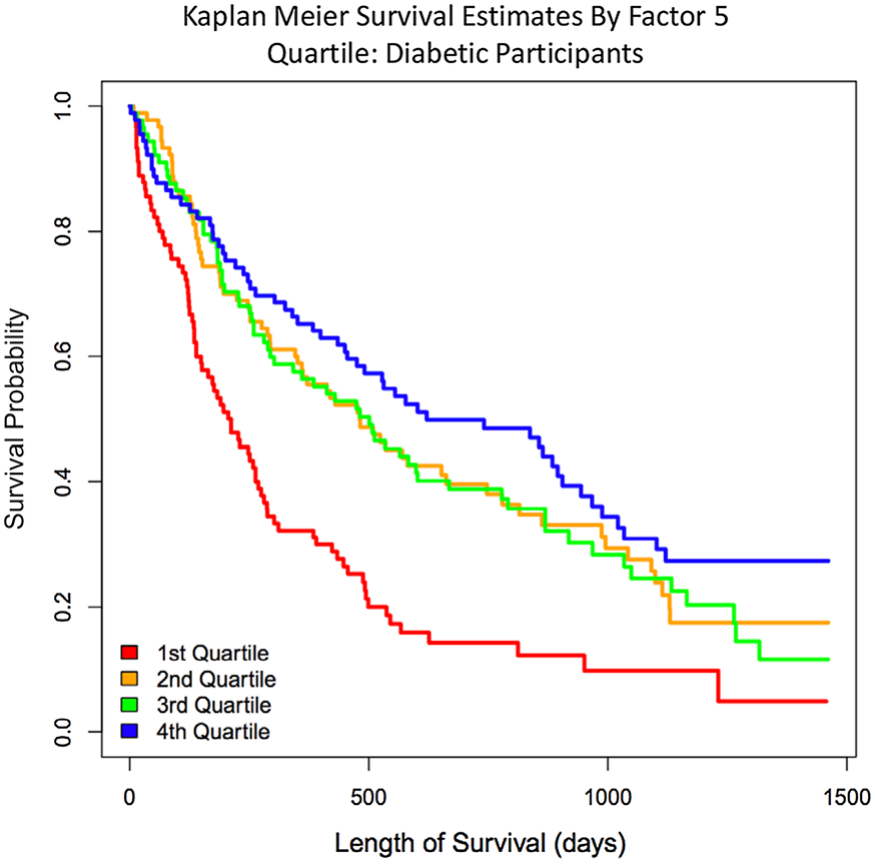
Kaplan–Meier curves illustrating time to all-cause mortality or all-cause hospitalization by quartile of metabolite factor 5 in diabetic participants

Table 4 displays the results of the Cox proportional hazard analysis for association between metabolite factors with time-to-event for the primary composite outcome of all-cause mortality or all-cause hospitalization. The median overall follow-up time for the entire cohort was 412.5 days: 412 for diabetic patients and 415 days for non-diabetic patients. In the overall cohort, factors 4, 5 and 8 were significantly associated with the primary composite endpoint of all-cause mortality or hospitalization after FDR adjustment for multiple comparisons in univariate analyses. Taking into consideration the directionality of the hazard ratio with direction of factor loadings, this shows that higher levels of individual SCDA metabolites (from factor 4), individual LCAC metabolites (from factor 5), and individual medium chain acylcarnitine and SCDA metabolites (from factor 8) are associated with a shorter time-to-event (i.e., greater risk). In multivariate models adjusted for race, sex, age, baseline LVEF, baseline creatinine, BMI, baseline peak VO_2_, BUN, CPX duration, and exercise treatment, only factor 5 (LCAC) remained significantly associated with time-to-event in the overall cohort (HR 0.80, p  =  8.22  ×  10^−8^; Fig. 1).

**Table 4:** Association of principal components factors with time to all-cause mortality or all-cause hospitalization

Factor	Overall				Non-diabetic			Diabetic			Interaction p value
	HR	p value		BH p value	HR	p value	BH p value	HR	p value	BH p value	
Unadjusted											
1	0.964	0.325	0.407								
2	1.020	0.602	0.602								
3	1.094	0.047	0.094								
4	**1.162**	**0.006**	**0.024**		**1.265**	**0.008**	**0.008**	1.097	0.186	0.279	0.244
5	**0.800**	**5E-11**	**6E-10**		**0.864**	**0.004**	**0.006**	**0.629**	**8E-11**	**2E-10**	**2E-4**
6	1.115	0.021	0.050								
7	0.896	0.018	0.050								
8	**0.884**	**0.004**	**0.024**		**0.783**	**0.001**	**0.003**	0.943	0.283	0.283	0.039
9	1.086	0.078	0.134								
10	1.042	0.373	0.407								
11	1.059	0.262	0.393								
12	1.041	0.365	0.407								
Adjusted^a^											
4	0.933	0.352	0.352		0.865	0.289	0.289	0.975	0.783	0.832	0.725
5	0.798	**2.74E-8**	**8.22E-8**		0.902	0.104	0.156	0.635	**3.21E-9**	**9.62E-9**	**0.001**
8	0.939	0.213	0.320		0.814	0.028	0.084	1.01	0.832	0.832	**0.012**

^a^Model is adjusted for race (white vs. non-white), sex, age, baseline left ventricular ejection fraction, creatinine, BMI, baseline peak VO_2_, treatment group, BUN and CPX duration

P-values in bold indicate statistically significant association

When significant factors from the overall cohort univariate analyses (factors 4, 5 and 8) were stratified by DM status in a priori defined analyses, all factors showed differences in effect sizes between participants with and without DM. For example, factor 5 was more significantly associated with time-to-event in participants with DM than in those without (HR 0.63, p  =  8  ×  10^−11^ v. HR 0.86, p  =  0.004). In formal analyses including an interaction term between metabolite factor level and DM, factor 5 was the only factor which showed significant interaction in both univariate (p  =  2.0  ×  10^−4^) and multivariate analyses (p  =  0.001).

Given these results, we sought to determine whether factor 5 changed differentially in participants with and without DM randomized to the exercise group. In these exploratory analyses, we found that there was a trend toward significance with factor 5 levels changing to a lesser extent in participants with DM after exercise training (mean ∆ 0.090, p  =  0.23) than in those without DM (mean ∆ 0.164, p  =  0.08).

### Individual LCAC results

Given our strongest results for both factor 1 (with pVO_2_ and for differential effects by DM status) and factor 5 (with incident events and differential outcomes by DM status), and the fact that both metabolite factors contain high metabolite loads for LCAC but also contain other metabolites, we then performed analyses of all individual metabolites with high factor loads in these factors (i.e., absolute value of factor load  >  0.4) to understand whether these factors represent a shared pathway or two different metabolic processes. These results confirmed the concordance of significant results for factor 1 and factor 5 as both being due to LCACs (and not because of other metabolites within those factors). Specifically, the LCAC C16, C16:1, C18, and C18:1 showed the largest effect sizes for association with pVO_2_ (Additional file 2: Table S2) and the LCAC C16, C18, C18:1 and C18:2 showed the largest effect sizes for time to event, particularly in diabetic participants (Additional file 2: Table S3). Interestingly, when time-to-event analysis was performed in a cohort of participants without HF from the CATHGEN study, these LCACs were not associated with all-cause mortality, both overall and in analyses stratified by diabetes (Additional file 2: Table S4). Absolute values of these individual metabolites stratified by the primary endpoint of all-cause mortality or hospitalization from the HF-ACTION cohort can be found in Additional file 2: Table S5. These results support the conclusion that, across pVO_2_ and time to event analyses, higher LCAC levels are associated with worse pVO_2_ and worse clinical outcomes especially in individuals with HF and DM.

## Discussion

Leveraging a unique study of exercise training in participants with HF with reduced ejection fraction with carefully adjudicated phenotypes including exercise capacity, we have found that metabolite signatures including long-chain acylcarnitines (LCAC) are associated with decreased exercise capacity and are predictive of incident adverse events even after adjustment for clinical covariates. Importantly, we also find that in patients with HF these metabolites show interactions with DM status with stronger negative associations with pVO_2_ in participants with DM, greater predictive capabilities for adverse events in participants with DM, and with a trend for changing to a lesser extent with exercise in participants with DM. Importantly, we did not observe any significant associations between LCAC and adverse incident outcomes in a group of non-HF participants with and without DM, suggesting that the prognostic implications of these fatty acid oxidation biomarkers are unique to patients with HF. Accumulation of circulating LCAC may reflect impaired mitochondrial fatty acid oxidation, both at the myocardial and skeletal muscle level, and identifies this molecular signature as a potentially important pathway in heart failure and diabetes that relates to clinical outcomes.

LCACs—long chain fatty acyl-CoAs which are esterified to carnitine—facilitate transfer of long-chain fatty acids into the mitochondria where they can be metabolized via beta oxidation [13]. Prior studies suggest that the metabolism of the healthy myocardium relies primarily on the oxidation of long-chain fatty acids to meet its significant energy demands. In the setting of DM, insulin resistance promotes free fatty acid uptake by cardiomyocytes, which can contribute to mitochondrial dysfunction, accumulation of lipotoxic and reactive oxygen species, and in turn impair fatty acid oxidation [14]. LCAC are intermediates of long-chain fatty acid oxidation, and in the setting of mitochondrial dysfunction can represent byproducts of incomplete fatty acid oxidation. We have previously shown that levels of circulating LCAC significantly increase across the spectrum of clinical heart failure, being lowest in healthy controls, intermediate in patients with heart failure with preserved ejection fraction, and highest in patients with heart failure with reduced ejection fraction [10]. In a prior sub-analysis of HF-ACTION patients with advanced HF, LCAC factors were associated with cardiovascular death and heart failure hospitalization [11]. Interestingly, several components of the LCAC factor, namely C16, C18:1, and C18:2, were markedly decreased after LVAD support, suggesting that reconstitution of cardiac output and improvement in skeletal muscle and end-organ perfusion may modulate systemic and myocardial bioenergetics [11]. While changes in fatty acid oxidation and LCACs have previously been implicated at the biochemical level in patients with HF, to our knowledge no prior studies have evaluated LCAC as a peripheral biomarker in concomitant HF and DM or examined their association with exercise capacity [15, 16]. Our results suggest that in the setting of the combination of DM and HF, impaired mitochondrial fatty acid oxidation, as reflected by accumulation of LCACs in the circulation, is at play, and that the decreased exercise capacity and outcomes seen in DM in our study could be due to the inability to effectively utilize fatty acids as a fuel source. Further, our results suggest that the effects of exercise on pVO_2_ may be attenuated in the setting of this dysregulated fatty acid oxidation in DM, providing a molecular context for the differential responses seen in the overall HF-ACTION clinical trial where participants in the treatment arm with DM experienced a smaller mean increase in pVO_2_ as compared to those without DM (p  =  0.03) [3].

Preclinical mouse models have also identified impaired long-chain fatty acid oxidation in HF and DM. These studies have demonstrated that insulin resistance promotes fatty acid uptake in cardiomyocytes by increasing translocation of CD36 to the plasma membrane, which in turn results in increased rate of fatty acid and triglyceride uptake [17, 18]. Translocation of the GLUT-4 glucose transporter is also downregulated, allowing less influx of glucose as fuel substrate. The increase in intra-myocardial fatty acids and triglycerides are thought to contribute to lipotoxicity and subsequent myocardial contractile dysfunction, identifying a plausible link between insulin resistance and systolic heart failure [19]. In confirmatory pathway interrogation, inhibition of CD-36 translocation has been shown to decrease intracellular lipid accumulation and subsequently prevent the development of myocardial dysfunction [20].

Down-regulation of fatty acid oxidation in the progression of HF has also been supported by myocardial transcriptomic data showing down-regulation of genes involved in cardiomyocyte fatty acid transport and oxidation with elevated myocardial LCACs in a mixed ischemic and pressure overload mouse model of HF [21]. Metabolic impairment in both the myocardium and peripheral organs has also been described in HF pathophysiology, where accumulation of LCACs may be reporting on this state of inefficient mitochondrial fatty acid oxidation [6, 22, 23]. Although data from preclinical animal and cell culture studies suggests that plasma levels of LCACs reflect primarily secretion from the liver and skeletal muscle, attempts to identify tissue-specific contributions to circulating plasma LCAC in humans as a result of metabolic impairment remains lacking [24]. At the level of the myocardium, increased LCACs cause electrophysiologic disturbances, inflammation, and cellular stress via reactive oxygen species and bioenergetic deficiency which translates into increased risk of arrhythmias, myocardial fibrosis, systolic dysfunction and ultimately clinical HF [25, 26]. There also exists clear association between elevated LCAC levels and skeletal muscle metabolism, which may be impaired in the settings of both HF and DM independently [27]. Taken together, these preclinical data and the results of our study support the hypothesis that LCACs report on impaired fatty acid oxidation at the level of both the skeletal muscle and myocardium and may be a marker, or mediator, of decreased peak VO_2_ in patients with DM and HF. Additional preclinical and translational work is needed to definitively identify tissue-specific substrate utilization and metabolic pathways that contribute to the elevated circulating levels of LCACs demonstrated here.

There are several strengths to our approach to identifying biomarkers of adverse incidenct events in concomitant HF and DM. First, we used samples collected as part of a carefully adjudicated clinical trial with both intermediate and hard outcomes. Further, the use of cardiopulmonary exercise testing with pVO_2_ as an accurate marker of exercise capability is a reproducible and reliable surrogate of aerobic capacity. We used a large number of metabolites that were measured in an accurate fashion with absolute quantification by virtue of addition of internal standards. However, limitations of the study should be noted. Most importantly, the source of circulating LCAC cannot be ascertained in this human study. Thus, while we hypothesize that the myocardium could be a source given the underlying HF phenotype, we recognize that skeletal muscle, in particular, likely plays a significant role in plasma LCAC accumulation. Similarly, ketone levels were not assayed in this particular cohort, which limits our insights into the interplay between LCAC and other fuel sources. Regardless of the tissue source of LCAC, however, we believe in the importance of recognizing these metabolites as peripheral biomarkers of important clinical outcomes even after adjustment for important covariates. Additionally, we were unable to adjust for metabolite batch in analyses due to unintentional consequences of our experimental design which confounded participant diabetes status with assay batch. Although we do not believe that batch is responsible for the signals we are seeing, we were unable to model their effects.

## Conclusion

In conclusion, using samples collected as part of a carefully conducted unique study of exercise in HF with a large proportion of participants with DM enabling a priori defined analyses of HF and DM, we have for the first time identified circulating LCAC metabolite levels as differentially associated with intermediate and hard outcomes in HF and DM. These results suggest that patients with the combination of DM and HF may have impaired ability to utilize long-chain fatty acids as a fuel source, resulting in bioenergetic disadvantage reflected in worse exercise capacity, worse long-term outcomes, and lesser response to exercise.

## Supplementary Information

**Additional file 1: Figure S1.** Change in C18 levels in treatment and control arm between baseline and 3-month timepoint.

**Additional file 2: Table S1.** Association of changes in pVO_2_ with baseline factors. **Table S2.** Individual metabolites and their association with pVO_2_. **Table S3.** Association of individual metabolites and time to primary clinical outcome (multivariate model). **Table S4.** Association of individual metabolites and all-cause mortality (time to event, multivariate model) in CATHGEN. **Table S5.** Absolute baseline metabolite concentrations stratified by primary outcome of all-cause mortality or hospitalization.

## Data Availability

The clinical datasets analyzed during the current study are available by request in the BIOLINCC repository.
