# Supplementary material for: Circulating long chain acylcarnitines and outcomes in diabetic heart failure: an HF-ACTION clinical trial substudy
Source: Cardiovasc Diabetol. 2021 Aug 3;20:161. doi: 10.1186/s12933-021-01353-z (PMC8336082; doi:10.1186/s12933-021-01353-z)
Supplement: Supplementary file 1 — Additional file 1: Figure S1. Change in C18 levels in treatment and control arm between baseline and 3-month timepoint. [file 12933_2021_1353_MOESM1_ESM.pptx]

## Slide 1
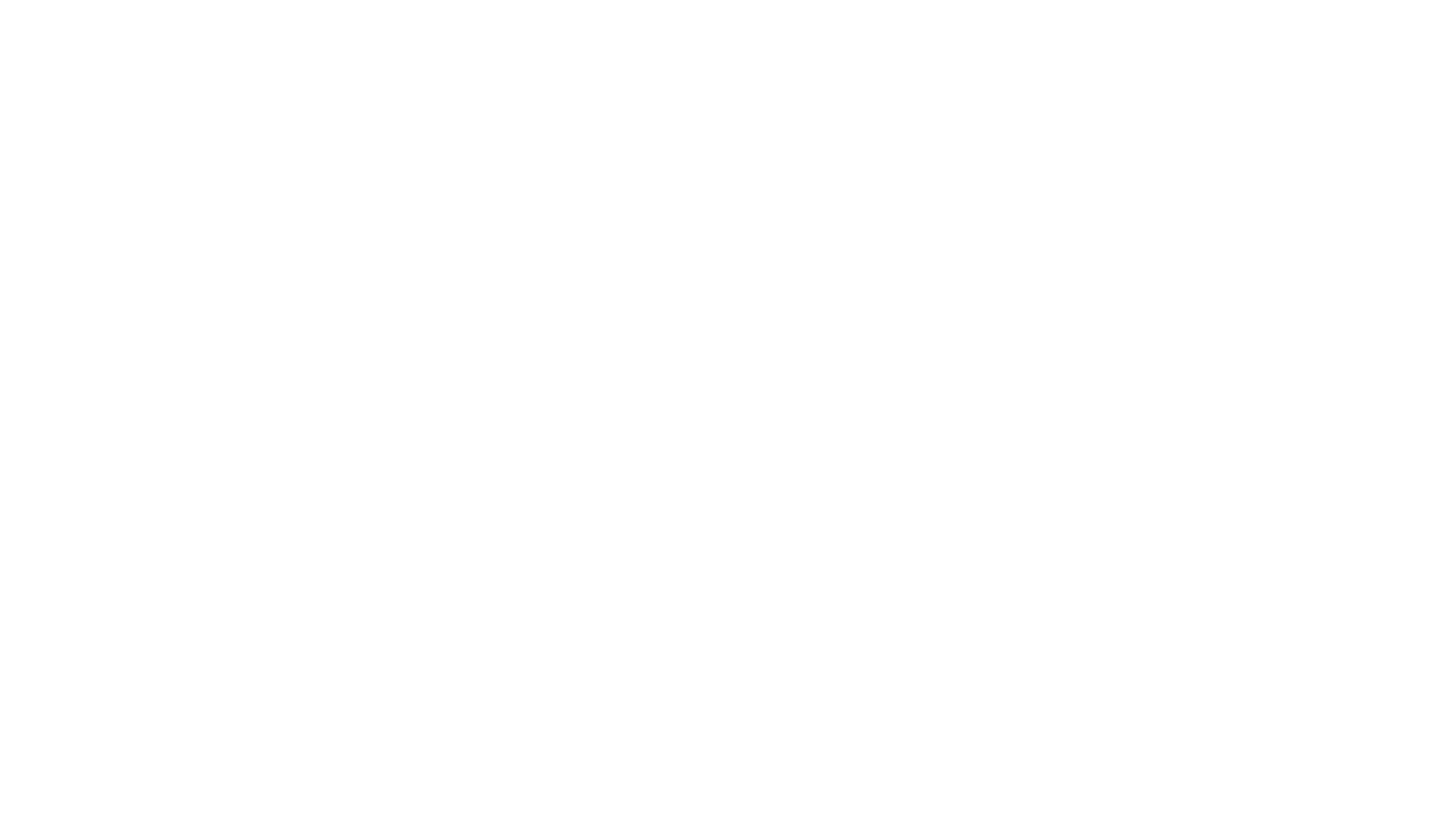

## Slide 2
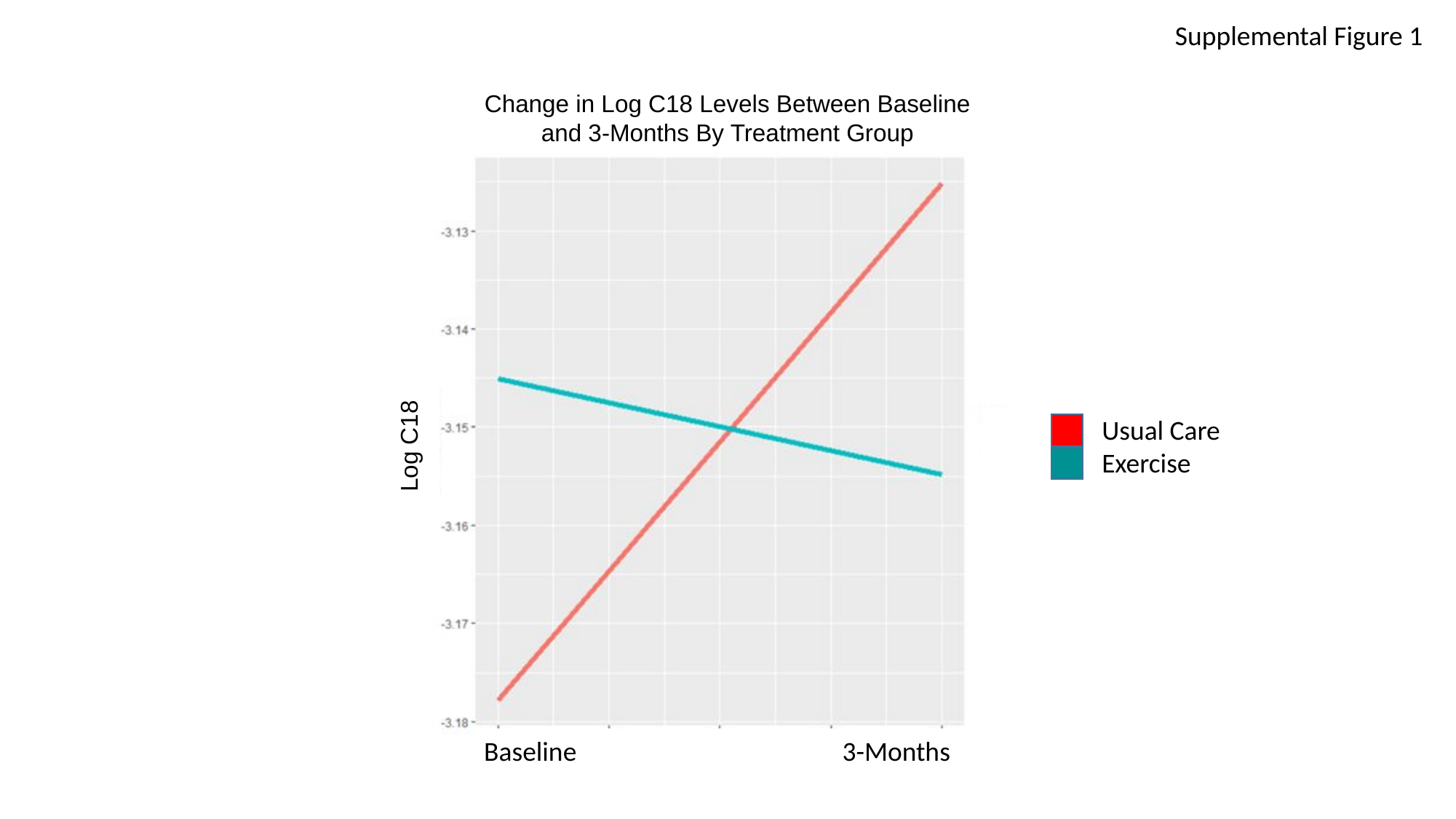

Supplemental Figure 1
Change in Log C18 Levels Between Baseline and 3-Months By Treatment Group
Log C18
	Usual Care
	Exercise
Baseline			 3-Months
