# Supplementary material for: Circulating long chain acylcarnitines and outcomes in diabetic heart failure: an HF-ACTION clinical trial substudy
Source: Cardiovasc Diabetol. 2021 Aug 3;20:161. doi: 10.1186/s12933-021-01353-z (PMC8336082; doi:10.1186/s12933-021-01353-z)
Supplement: Supplementary file 2 — Additional file 2: Table S1. Association of changes in pVO2 with baseline factors. Table S2. Individual metabolites and their association with pVO2. Table S3. Association of individual metabolites and time to primary clinical outcome (multivariate model). Table S4. Association of individual metabolites and all-cause mortality (time to event, multivariate model) in CATHGEN. Table S5. Absolute baseline metabolite concentrations stratified by primary outcome of all-cause mortality or hospitalization. [file 12933_2021_1353_MOESM2_ESM.docx]

**Supplemental Table 1: Association of Changes in pVO_2_ with Baseline Factors**

| **Factor­** | **Description** | **Beta** | **p-value** | **BH p-value** |
| --- | --- | --- | --- | --- |
| 1 | Long Chain Acylcarnitines | 0.126 | 0.202 | 0.601 |
| 2 | Long Chain Dicarboxylacylcarnitines | -0.178 | 0.086 | 0.370 |
| 3 | Short Chain Acylcarnitines | 0.052 | 0.611 | 0.815 |
| 4 | Medium Chain Dicarboxylacylcarnitines | -0.143 | 0.269 | 0.601 |
| 5 | Long Chain Acylcarnitines | 0.041 | 0.695 | 0.834 |
| 6 | Amino Acids (Gly, Ser, Arg) | -0.066 | 0.506 | 0.815 |
| 7 | Miscellaneous | -0.017 | 0.866 | 0.866 |
| 8 | Medium Chain Acylcarnitines | 0.181 | 0.070 | 0.370 |
| 9 | Amino Acids ( Ala, Pro, His) | -0.024 | 0.807 | 0.866 |
| 10 | Amino Acids ( His(+), Asx(-), Arg(+)) | 0.168 | 0.092 | 0.370 |
| 11 | Long Chain Dicarboxylacylcarnitines | 0.107 | 0.300 | 0.601 |
| 12 | Branched Chain Amino Acids \| Amino Acids ( Val, Leu/Ile, Phe, Tyr, Orn) | 0.058 | 0.568 | 0.815 |

**Supplemental Table 2: Individual Metabolites and Their Association With pVO_2_**

| **Metabolite** | **Factor 1 Loading** | **Metabolite Beta** | **Metabolite p-value** | **Diabetes**  **Beta** | **Diabetes**  **p-value** | **No Diabetes Beta** | **No Diabetes**  **p-value** |
| --- | --- | --- | --- | --- | --- | --- | --- |
| C8 | -0.92 | -2.231 | 3E-15 | -2.518 | 7E-12 | -1.928 | 5E-6 |
| C10:1 | -0.75 | -2.921 | 1E-18 | -3.237 | 1E-13 | -2.391 | 1E-6 |
| C10 | -0.95 | -1.882 | 1E-11 | -1.832 | 1E-07 | -2.193 | 7E-7 |
| C12:1 | -0.66 | -2.609 | 6E-16 | -2.876 | 7E-12 | -2.441 | 4E-7 |
| C12 | -0.94 | -2.609 | 3E-16 | -2.979 | 2E-12 | -2.524 | 7E-8 |
| C14:2 | -0.83 | -2.529 | 2E-18 | -2.474 | 2E-11 | -2.526 | 1E-8 |
| C14:1 | -0.84 | -2.868 | 4E-20 | -3.07 | 4E-14 | -2.644 | 1E-8 |
| C14 | -0.89 | -2.209 | 2E-11 | -1.793 | 3E-6 | -3.148 | 8E-8 |
| **C16** | **-0.60** | **-4.093** | **2E-14** | **-4.245** | **8E-10** | **-3.679** | **7E-6** |
| C18:2 | -0.42 | -2.411 | 3E-10 | -2.838 | 2E-9 | -2.066 | 0.001 |
| **C18:1** | **-0.44** | **-3.074** | **4E-12** | **-3.591** | **2E-10** | **-2.826** | **3E-5** |
| **C18** | **-0.49** | **-2.991** | **8E-08** | **-2.868** | **1E-4** | **-2.824** | **0.001** |
| C16:2 | -0.83 | -0.327 | 0.002 | -0.324 | 0.004 | -0.899 | 2E-4 |
| **C16:1** | **-0.83** | **-2.984** | **7.E-20** | **-2.738** | **5E-11** | **-3.047** | **2E-9** |

**Supplemental Table 3: Association of Individual Metabolites and Time to Primary Clinical Outcome (Multivariate Model)**

| **Metabolite** | **Metabolite HR** | **Metabolite p-value** | **Diabetic**  **HR** | **Diabetic**  **p-value** | **Non-Diabetic HR** | **Non-Diabetic**  **p-value** |
| --- | --- | --- | --- | --- | --- | --- |
| Arg | 0.568 | 6.90E-4 | 0.424 | 5.12E-5 | 0.816 | 0.465 |
| C8 | 1.049 | 0.608 | 1.247 | 0.077 | 0.825 | 0.195 |
| C10:1 | 1.15 | 0.236 | 1.407 | 0.041 | 0.894 | 0.519 |
| C10 | 1.084 | 0.375 | 1.399 | 0.009 | 0.763 | 0.075 |
| C12:1 | 1.237 | 0.058 | 1.505 | 0.008 | 0.969 | 0.851 |
| C12 | 1.132 | 0.236 | 1.524 | 0.007 | 0.830 | 0.267 |
| C14:2 | 1.097 | 0.346 | 1.318 | 0.045 | 0.860 | 0.326 |
| C14:1 | 1.110 | 0.354 | 1.337 | 0.075 | 0.912 | 0.586 |
| C14 | 1.215 | 0.078 | 1.643 | 0.003 | 0.840 | 0.381 |
| **C16** | **1.560** | **0.013** | **2.350** | **5.05E-4** | 1.010 | 0.972 |
| **C18:2** | **1.575** | **1.94E-4** | **2.026** | **5.77E-05** | 1.115 | 0.562 |
| **C18:1** | **1.815** | **4.67E-6** | **2.472** | **1.49E-05** | 1.229 | 0.351 |
| **C18** | **1.363** | **0.070** | **2.235** | **0.001** | 0.796 | 0.395 |
| C16:2 | 1.055 | 0.091 | 1.054 | 0.146 | 1.018 | 0.816 |
| **C16:1** | **1.277** | **0.026** | **1.436** | **0.017** | 1.125 | 0.505 |
| **C20:4** | **1.213** | **0.005** | **1.247** | **0.004** | 1.105 | 0.579 |

**Supplemental Table 4: Association of Individual Metabolites and All-Cause Mortality(Time to Event, Multivariate Model) in CATHGEN**

| **Metabolite** | **Metabolite HR** | **Metabolite p-value** | **Diabetic**  **HR** | **Diabetic**  **p-value** | **Non-Diabetic HR** | **Non-Diabetic p-value** |
| --- | --- | --- | --- | --- | --- | --- |
| Arg | 0.82 | 0.15 | 0.83 | 0.45 | 0.84 | 0.33 |
| C8 | 1.19 | 0.042 | 1.22 | 0.22 | 1.19 | 0.12 |
| C10:1 | 1.44 | 0.001 | 1.38 | 0.12 | 1.46 | 0.005 |
| C10 | 1.19 | 0.031 | 1.18 | 0.24 | 1.19 | 0.08 |
| C12:1 | 1.47 | <0.001 | 1.37 | 0.12 | 1.44 | 0.004 |
| C12 | 1.23 | 0.04 | 1.36 | 0.10 | 1.17 | 0.19 |
| C14:2 | 1.23 | 0.01 | 1.29 | 0.10 | 1.25 | 0.03 |
| C14:1 | 1.22 | 0.03 | 1.23 | 0.22 | 1.25 | 0.04 |
| C14 | 1.04 | 0.66 | 0.96 | 0.84 | 1.10 | 0.37 |
| C16 | 1.03 | 0.87 | 1.48 | 0.18 | 0.93 | 0.70 |
| C18:2 | 1.23 | 0.07 | 1.38 | 0.13 | 1.19 | 0.19 |
| C18:1 | 1.23 | 0.11 | 1.33 | 0.24 | 1.19 | 0.27 |
| C18 | 1.16 | 0.17 | 1.31 | 0.18 | 1.09 | 0.53 |
| C16:2 | 1.03 | 0.53 | 1.01 | 0.88 | 1.04 | 0.45 |
| C16:1 | 1.19 | 0.11 | 1.35 | 0.15 | 1.14 | 0.32 |
| C20:4 | 0.97 | 0.43 | 1.06 | 0.34 | 0.94 | 0.10 |

**Supplemental Table 5: Absolute Baseline Metabolite Concentrations Stratified by Primary Outcome of All-Cause Mortality or Hospitalization**

| **Metabolite** | **Controls** | **Cases** |
| --- | --- | --- |
| Arg | 75.9230 [64.1151, 92.2206] | 71.5069 [60.6616, 86.3560] |
| C8 | 0.1207 [0.0864, 0.1731] | 0.1353 [0.0939, 0.1886] |
| C10:1 | 0.1599 [0.1227, 0.1985] | 0.1819 [0.1290, 0.2437] |
| C10 | 0.2272 [0.1738, 0.3210] | 0.2550 [0.1911, 0.3391] |
| C12:1 | 0.0973 [0.0670, 0.1418] | 0.1171 [0.0843, 0.1550] |
| C12 | 0.0752 [0.0566, 0.1004] | 0.0824 [0.0603, 0.1153] |
| C14:2 | 0.0353 [0.0266, 0.0479] | 0.0397 [0.0283, 0.0561] |
| C14:1 | 0.0661 [0.0488, 0.0910] | 0.0747 [0.0548, 0.1007] |
| C14 | 0.0292 [0.0235, 0.0378] | 0.0325 [0.0254, 0.0416] |
| C16 | 0.0738 [0.0614, 0.0871] | 0.0795 [0.0664, 0.0974] |
| C18:2 | 0.0441 [0.0352, 0.0569] | 0.0511 [0.0387, 0.0726] |
| C18:1 | 0.0976 [0.0797, 0.1281] | 0.1129 [0.0877, 0.1431] |
| C18 | 0.0397 [0.0348, 0.0460] | 0.0432 [0.0357, 0.0531] |
| C16:2 | 0.0058 [0.0024, 0.0100] | 0.0065 [0.0026, 0.0111] |
| C16:1 | 0.0179 [0.0135, 0.0228] | 0.0198 [0.0146, 0.0262] |
| C20:4 | 0.0057 [0.0035, 0.0094] | 0.0068 [0.0049, 0.0110] |

*All units are uM.
